# Pneumatic displacement and intravitreal bevacizumab for management of submacular hemorrhage in choroidal neovascular membrane

**DOI:** 10.4103/0301-4738.60078

**Published:** 2010

**Authors:** Manisha Agarwal, S P Chaudhary, Ritesh Narula, Simpy Rajpal

**Affiliations:** Vitreoretina Services, Dr. Shroff's Charity Eye Hospital, 5027, Kedar Nath Road, Daryaganj, New-Delhi, India

Dear Editor,

We read with great interest the article titled “Pneumatic displacement and intravitreal bevacizumab: A new approach for management of submacular hemorrhage in choroidal neovascular membrane” by Chawla S *et al*.[1]

We would like to share our experience of a 52-year-old male patient who presented with blurring of vision in the right eye for the last one month and a sudden drop of vision with a central black spot of two days duration. The best corrected visual acuity (BCVA) was counting fingers close to face in the right eye and counting fingers at 4 meters in the left eye. Intraocular pressure and slit-lamp examination were within normal limits. Fundus examination of the right eye showed submacular hemorrhage with breakthrough vitreous hemorrhage [Fig. 1] and the left eye was normal. He was diagnosed to have anisometropic amblyopia in the left eye. Fundus fluorescein angiography (FFA) of the right eye showed blocked fluorescence corresponding to submacular hemorrhage. He underwent gas injection with tissue plasminogen activator (TPA) (50 ug/0.05 ml)[2] under topical anesthesia and aseptic precautions. This was followed by prone positioning of the patient.

**Figure 1:**
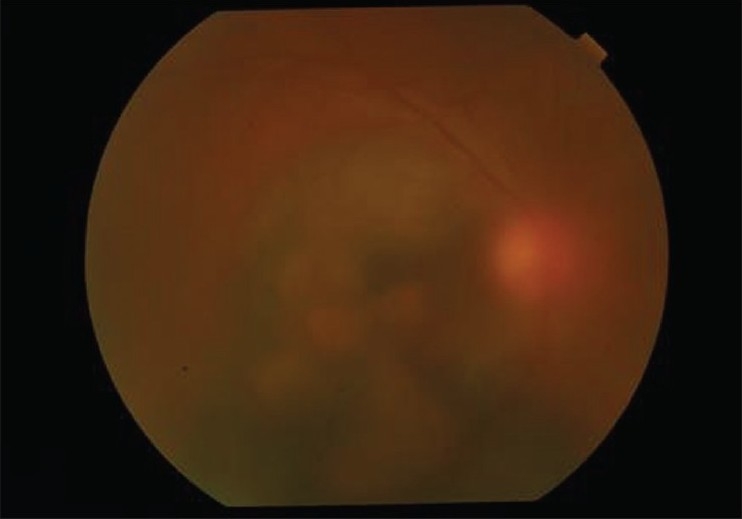
Color fundus photograph of the right eye showing extensive submacular hemorrhage with breakthrough vitreous hemorrhage

Fundus examination of the right eye after one week showed displacement of the submacular hemorrhage inferiorly [Fig. 2]. FFA of the right eye showed an active extrafoveal choroidal neovascular membrane (CNVM) as the source of the hemorrhage with blocked fluorescence inferiorly corresponding to the displaced submacular hemorrhage [Figs. 3–6]. Intravitreal bevacizumab injection was given in dose (1.25 mg/0.05 ml) under topical anesthesia and strict aseptic precautions. He subsequently underwent three repeat intravitreal injections of bevacizumab in the right eye.

**Figure 2:**
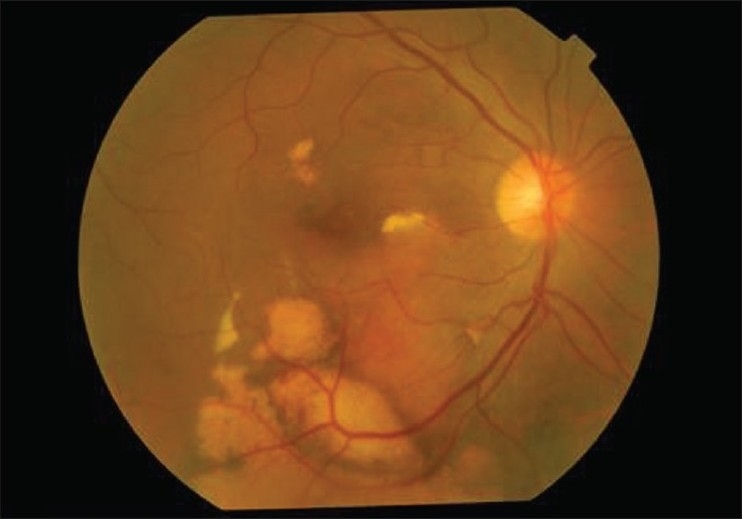
Color fundus photograph of the right eye after gas tamponade and intravitreal tissue plasminogen activator injection showing displacement of the submacular hemorrhage

**Figures 3-6:**
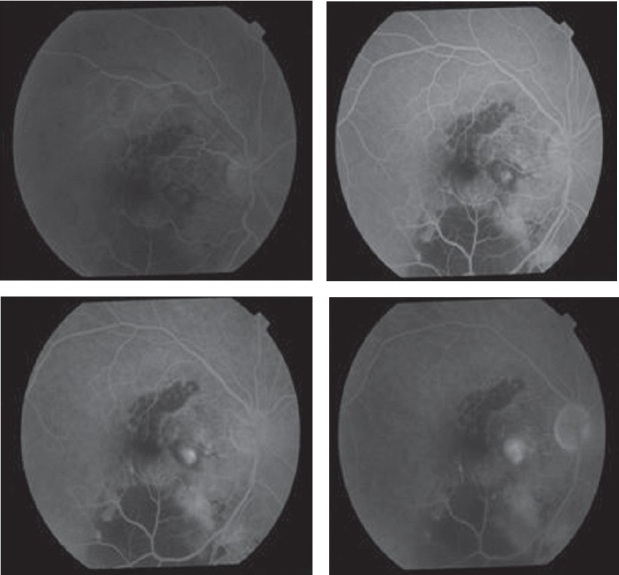
Fundus fluorescein angiograms of the right eye showing an actively leaking extrafoveal choroidal neovascular membrane as the source of submacular hemorrhage

At nine months follow-up the BCVA in the right eye was 20/120P, N36. Fundus examination showed old sub-retinal hemorrhage displaced to the lower macular area. FFA of the right eye showed a scarred CNVM with blocked fluorescence in the inferior macular area corresponding to the persistent old hemorrhage. Optical coherence tomography of the right eye showed a normal foveal contour and the scarred extrafoveal CNVM.

Management of the cases with submacular hemorrhage secondary to CNVM are often challenging. Various treatment options are available for the submacular hemorrhage such as pneumatic displacement with or without TPA or vitrectomy with evacuation of the blood. Chawla *et al*.,[1] in their case series experienced a good visual recovery in all the four cases. However the visual recovery in our case was not good even after a good displacement of the submacular hemorrhage.

As mentioned in the literature, the visual prognosis in cases with submacular hemorrhage depends on a number of factors such as location of the CNVM (extrafoveal, subfoveal or juxtafoveal), duration of the submacular hemorrhage - longer the duration worse is the prognosis due to photoreceptor damage, level of the hemorrhage -sub-retinal or sub-retinal pigment epithelial (RPE), the size and the thickness of the hemorrhage and lastly the underlying disease process.[2]

The combination of gas tamponade with an anti-vascular endothelial growth factor (VEGF) such as bevacizumab without using TPA does provide an inexpensive treatment option to our patients, however, the visual recovery may not always be predictable.

## References

[1] Chawla S, Misra V, Khemcandani M (2009). Pneumatic displacement and intravitreal bevacizumab: A new approach for management of submacular hemorrhage in choroidal neovascular membrane. Indian J Ophthalmol.

[2] Tennant TS, Borrillo JL, Regillo CD (2002). Management of submacular hemorrhage. Ophthalmol Clin N Am.

